# Investigating the roles of age, sex, depression, and anxiety for valence and arousal ratings of words: a population-based study

**DOI:** 10.1186/s40359-020-00485-3

**Published:** 2020-11-07

**Authors:** Henning Teismann, Johanna Kissler, Klaus Berger

**Affiliations:** 1 Institute of Epidemiology and Social Medicine, University of Münster, Albert-Schweitzer-Campus 1 (Building D3), 48149 Münster, Germany; 2 Department of Psychology, University of Bielefeld, Bielefeld, Germany; 3 Center of Excellence Cognitive Interaction Technology (CITEC), University of Bielefeld, Bielefeld, Germany

**Keywords:** Affect, Emotion, Affective quality perception, Valence, Arousal, Unpleasant, Neutral, Pleasant, Words, BiDirect Study, Population-based, Observational

## Abstract

**Background:**

The perception of the affective quality of stimuli with regard to valence and arousal has mostly been studied in laboratory experiments. Population-based research may complement such studies by accessing larger, older, better balanced, and more heterogeneous samples. Several characteristics, among them age, sex, depression, or anxiety, were found to be associated with affective quality perception. Here, we intended to transfer valence and arousal rating methods from experimental to population-based research. Our aim was to assess the feasibility of obtaining and determining the structure of valence and arousal ratings in the setting of the large observational BiDirect Study. Moreover, we explored the roles of age, sex, depression, and anxiety for valence and arousal ratings of words.

**Methods:**

704 participants provided valence and arousal ratings for 12 written nouns pre-categorized as unpleasant, neutral, or pleasant. Predictors of valence and arousal ratings (i.e. age, sex, depression, and anxiety) were analyzed for six outcomes that emerge by combining two affective dimensions with three words categories. Data were modeled with multiple linear regression. Relative predictor importance was quantified by model-explained variance decomposition.

**Results:**

Overall, average population-based ratings replicated those found in laboratory settings. The model did not reach statistical significance in the valence dimension. In the arousal dimension, the model explained 5.4% (unpleasant), 4.6% (neutral), and 3.5% (pleasant) of the variance. (Trend) effects of sex on arousal ratings were found in all word categories (unpleasant: increased arousal in women; neutral, pleasant: decreased arousal in women). Effects of age and anxiety (increased arousal) were restricted to the neutral words.

**Conclusions:**

We report results of valence and arousal ratings of words in the setting of a large, observational, population-based study. Method transfer yielded acceptable data quality. The analyses demonstrated small effects of the selected predictors in the arousal dimension.

## Background

What emotions are and where they come from continues to be a matter of considerable debate. For example, one classical view holds that emotions can be regarded as discrete categories (i.e. anger, disgust, fear, happiness, sadness, and surprise [1]), which are built-in from birth and have universal “fingerprints” in the face, body, and brain. By contrast, a recent constructionist view conceptualizes emotions as complex, highly variable, context-, and goal-specific mental constructions (for recent reviews, see [2, 3]).

Emotions can be characterized as affective states. Affect refers to the subjective experience of emotions, moods, and other feelings [4]. The term affect essentially describes the basic sense of feeling, which ranges from pleasant to unpleasant and from agitated to calm [3]. According to the Circumplex Model of Affect [5, 6], both core affect (a subjective property: subjective feeling) and affective quality (a property of a stimulus: the potential that a stimulus has to change core affect) can be characterized by a certain degree of arousal (sense of energy: activation vs. quiescence) together with a certain degree of valence (hedonic tone: pleasure vs. displeasure). In this framework, valence is measured using a bipolar scale (unpleasant–neutral–pleasant), while arousal is measured with a unipolar scale (low–high) [7, 8]. Other dimensional models of affect (for a review, see e.g. [9]) vary in several aspects (e.g. the number and the labeling of the dimensions), but these models basically converge to identify valence and arousal as fundamental components of affect [4]. To measure valence and arousal, different strategies have been applied, among them e.g. observational techniques, or (neuro-)physiological recordings. However, it has been argued that structured self-reports (e.g. by means of numerical ratings scales) currently are the most feasible way to access these subjective dimensions [4, 10]. Noteworthy, several possibilities regarding the relationship between valence and arousal have been proposed [4]. The evidence available so far indicates that high arousal is more likely to go along with either high positive or high negative valence [4, 7, 11], so that the relationship between bipolar valence and unipolar arousal ratings usually reflects an inverted V shape. However, there is also great inter-individual variability [4, 11]. A recent paper [12] suggested that the relationship between valence and arousal may be conditional on the uncertainty (“ambiguity”) of perceived valence.

Age, sex, and traits or states of depression or anxiety are among the relevant factors which influence how humans experience affect or perceive affective quality. To begin with, it seems that emotional (compared to cognitive) functions are generally characterized by less decline during normal aging (for reviews, see [13, 14]). However, there appear to be specific changes: one prominent example among age-related findings regarding emotional functioning is the so-called age-related positivity effect [15, 16], which describes the observation that older adults tend to focus more on pleasant and less on unpleasant stimuli. For example, studies investigating memory for emotional pictures [17] or emotional words [18] found that older adults appear to selectively remember larger percentages of positive stimuli and smaller percentages of negative stimuli compared to younger adults. Regarding the role of sex for affective experience or affective quality perception, it has been reported that women may be more emotionally expressive and recognize emotions better and more automatically than men [19–22]. There is also evidence indicating that age [23–27] and sex [26–29] may affect valence and arousal ratings of words. However, given that previous results regarding age and sex were mixed and that effects tended to be weak [23, 26, 29], it seems worthwhile to further explore these effects in different settings.

Furthermore, states of depression and anxiety are characterized by marked dysfunctions of affective experience and affective quality perception; this includes the perception of the affective quality of words [30]. States of depression and anxiety have also been shown to modulate neural responses to affective stimuli such as faces [31–33], pictures [34], speech [35], and also words [36–41]. Depression patients in particular show more elaboration of negatively toned information, have trouble to disengage from negatively toned information, and show deficient cognitive control during the processing of negatively toned information [42]. According to the extended tripartite model of depression and anxiety [43], depression is characterized by increased negative and decreased positive affect, whereas anxiety is mostly identified by increased arousal. Thus, a separation of effects of depression and anxiety with regard to affective quality perception seems desirable.

Experimental research on affective quality perception has often utilized pictorial stimuli (e.g. [44]) depicting either evocative objects and scenes (including e.g. human or animal attack or mutilations) or facial expressions of emotion (e.g. anger or happiness) [45]. However, electrophysiological and hemodynamic studies demonstrated amplified cortical responses also during reading of words with emotional connotation/ content [46, 47], and changes in clinical populations have been demonstrated. For instance, a study using functional magnetic resonance imaging found that both unpleasant words (e.g. pain, catastrophe, victim) and pleasant words (e.g. love, baby, holidays) activated the amygdala. Unpleasant-word processing revealed a positive correlation between amygdala activity and scores of trait anxiety and subclinical depression. When comparing unpleasant word reading and neutral word reading, subjects with high trait anxiety also exhibited stronger functional coupling between left amygdala and the left dorso-lateral prefrontal cortex. These findings imply for example a modulation of unpleasant-word processing by subclinical depression and anxiety, as well as possible prefrontal compensatory processes during unintentional emotion regulation in subjects with higher trait anxiety [30]. Notably, word stimuli are easy to apply, and isolated unpleasant words are typically less disturbing than their pictorial counterparts, although responses induced by words in non-native speakers need to be regarded with caution [48, 49].

The first established database for affective words was Affective Norms for English Words (ANEW) [50]. Meanwhile, several databases of affective words have been compiled [28, 51–53], including some for German (e.g. [46, 54–56]). These databases included ratings of valence and arousal. However, the methodology differed between studies. For example, Kissler et al. [46] and Kanske et al. [54] used a bipolar nine-point pictorial Self-Assessment Manikin (SAM) scale [57] for valence and a unipolar nine-point SAM scale for arousal. Võ et al. [55] and Schmidtke et al. [56] used a seven-point (− 3 to 3) bipolar valence scale with verbal anchors, and a five-point SAM scale for arousal. The mere fact that different scales were applied complicates the comparison of ratings between studies. Moreover, the participants who rated the words differed with regard to age and sex distributions between studies.

Previous valence and arousal investigations (e.g. [23, 58, 59]) were typically executed as controlled laboratory experiments with a comparatively limited number of participants (≤ 100 overall, ~ 20 per age group). Population-based research has the potential to complement such laboratory findings, for example through accessing much larger, more heterogeneous (e.g. in terms of socio-demographic background), older, and better balanced samples (e.g. with regard to the sex, given that typical psychology undergraduate samples are predominately female). This may improve the generalizability of the findings, an issue which has been subject to recent debate in psychology (e.g. [60]). However, large population-based studies are typically characterized by less controlled conditions, including e.g. less-specifically trained and more frequently alternating personnel, or limited time to complete many different assessments, so that it is not self-evident that procedures that work well in the laboratory setting smoothly scale to the observational setting.

In the present project, we collected valence and arousal ratings of words within the setting of the BiDirect Study [61, 62]. The BiDirect Study is a large, population-based, observational, longitudinal cohort study originally designed to investigate the bidirectional relationship between depression and (subclinical) arteriosclerosis. BiDirect integrates three cohorts of adults aged 35–65 years at the time of recruitment: (1) patients with depression, (2) patients with cardiovascular disease due to arteriosclerosis, and (3) community-dwelling control subjects randomly invited from the registry of the city of Münster, Germany. Our goal was to assess the feasibility of obtaining and determining the structure of valence and arousal ratings in the population-based cohort of the BiDirect control subjects, which includes more than 900 individuals. Specifically, we aimed to elucidate the roles of demographic and clinical variables in influencing word ratings in this sample. Previous laboratory studies (e.g. [23, 24, 59]) have addressed the effect of age on word ratings for several hundred words, however with only about 20 participants per age bin. These studies revealed similarities across age groups, but also pointed to subtle differences. To our knowledge, none of these studies assessed the influence of sex, depression, or anxiety specifically for subjective ratings of valence and arousal in words. To this end, we examined the relative importance of age, sex, depression, and anxiety for the valence and arousal ratings of 12 words shown to differ highly in valence and arousal as well as associated neurophysiology in previous studies [30, 46]. Relative importance was quantified in terms of variance explained by the variables. These variables were chosen (1) because they had been found to be associated with affective quality perception in previous studies; moreover, (2) these variables are of specific interest in the framework of the BiDirect Study; and finally, (3) these variables are of general interest in many observational studies of mental (and also physical) health.

## Methods

### Stimuli

The nouns that were used in the present study to investigate valence and arousal ratings constitute a subset of the words introduced by Kissler et al. [46]. These words were pre-categorized into (1) high-arousing pleasant words, (2) low-arousing neutral words, and (3) high-arousing unpleasant words.

**Fig. 1:**
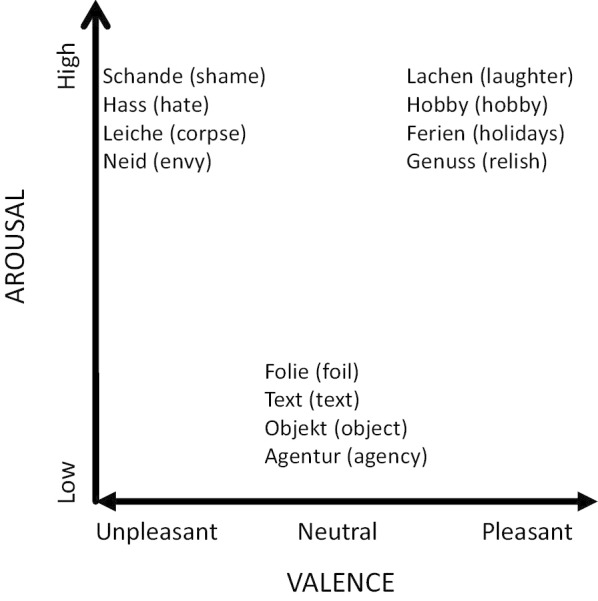
Mapping of the 12 word stimuli to three word categories in valence—arousal space (i.e. unpleasant—high, neutral—low, pleasant—high)

To choose a subset of the words from Kissler et al. [46] to be used here, we conducted a pretest at two study centers with 30 nouns and 15 adjectives in a convenience sample of 52 native German speakers, whose age distribution resembled that of the BiDirect Study participants (i.e. 35–65 years) in order to evaluate whether the pattern of valence and arousal pre-categorizations could be reproduced. Based on the results, we selected 12 nouns. Across word categories, the 12 nouns were comparable regarding concreteness, word length (in letters), and word frequency (per million), the latter based on counts for written German according to the CELEX database [63]. Figure 1 depicts schematically how the 12 words map to three word categories in (bipolar) valence and (unipolar) arousal spaces. The affective quality of the stimuli as represented by the three word categories expresses average estimates acquired under standard conditions [7].

During the baseline examination of the BiDirect Study, the valence and arousal ratings were gathered at the end of the neuropsychological assessment module, which included several tests of e.g. verbal learning and memory, attention, and executive functions. The 12 selected nouns were presented to all participants. With a short introductory text explaining the individuality of emotional experience, it was re-emphasized that the personal perception was of interest, and that there was no right or wrong. Written instructions were provided for both tasks. A bipolar nine-point rating scale (verbally labelled “very unpleasant” at the left end, “neither unpleasant nor pleasant” in the center, and “very pleasant” at the right end) quantified the perceived valence for each presented word. Another (unipolar) nine-point rating scale (labelled “not arousing” at the left end and “very arousing” at the right end) was used to assess the perceived arousal for each individual noun. The words, in each case together with a scale, were provided on two sheets of paper (one for the valence ratings, which had to be given first, and another one for the arousal ratings, which had to be given afterwards). A study nurse was present during the assessment to answer potential questions of the participants. The assessment took place in a quiet room. The whole procedure lasted about five minutes. Noteworthy, during the BiDirect Study the same 12 nouns were also used in a learning and memory task, which was conducted approximately 15 min prior to the valence and arousal ratings.

### Participants

Participants included into the present analyses were those from the population-based control cohort of the BiDirect Study [61, 62]. The BiDirect control cohort includes N = 911 subjects at baseline. For the current analyses, we excluded (1) subjects displaying pronounced difficulties regarding the German language (since several word stimuli were rather abstract and of relatively low frequency, and because the pretest had included only native German speakers), (2) subjects exhibiting missing values in variables that were used to derive predictor variables, and (3) subjects exhibiting missing values in further predictor or outcome variables that were used during analyses. After exclusion, 704 subjects remained to be analyzed.

### Selection of variables for analysis

To address the possibility that effects would differ between valences, the three word categories were analyzed separately. The six (continuous) outcome variables used here were the average valence or arousal ratings with respect to the unpleasant versus neutral versus pleasant word categories (i.e. valence—unpleasant, valence—neutral, valence—pleasant, and arousal—unpleasant, arousal—neutral, arousal—pleasant).

Explanatory variables of interest were age (continuous), sex, depression (categorical; no vs. yes), and anxiety (categorical; no vs. yes). Depression was “yes” if (1) the participant had reported a physician-assigned diagnosis of depression, or if (2) either MINI [64] item “A1” or MINI item “A2” was answered “yes”. Anxiety was “yes” if (1) the participant had reported a physician-assigned diagnosis of anxiety disorder, or if (2) MINI item “O1a” was answered “yes”. The MINI international neuropsychiatric interview [64] is a short structured clinical interview, which enables researchers to make diagnoses of psychiatric disorders according to DSM-IV or ICD-10. The MINI was originally designed for use in epidemiological studies and multicenter clinical trials.

In addition, we adjusted all analyses for educational attainment of the participants (categorical; level-1 = “certificate of secondary education or lower”; level-2 = “general certificate of secondary education”; level-3 = “university entrance diploma or vocation diploma”; level-4 = “university degree”) and for the examiners who had instructed participants.

All variables used during analyses were selected or derived in a purely theory-driven manner prior to analysis. No variable selection algorithms were applied.

### Analysis details

Data were analyzed with R using RStudio Desktop (RStudio Inc., Boston, MA, USA) with the objective to describe the relative importance of the explanatory variables of interest (i.e. age, sex, depression, and anxiety) with regard to the six different outcomes. The significance threshold was set to α = 0.05. All analyses should be regarded as exploratory.

First, we computed separate multiple linear regression models for the six outcomes. In a second step, in order to assess relative importance, we computed the (game-theory-based) LMG relative importance metric (developed by Lindemann, Merenda, and Gold [65]) based on the six linear models. LMG provides a decomposition of the model-explained variance into non-negative contributions. During LMG computation, R^2^ is partitioned by averaging over orders, i.e. variable order independence of the variance decomposition is guaranteed [65–67]. The LMG metric was bootstrapped using the contributed R package “relaimpo version 2.2–3” [68]. During the LMG computation, the explanatory variables of interest (i.e. age, sex, depression, and anxiety) were grouped, and it was adjusted for the potentially confounding variables (i.e. education and examiner). The measure of relative importance that we wished to interpret was the proportion of variance that was explained by a single explanatory variable of interest compared to the proportions of variance that were explained by the other explanatory variables of interest (stratified by outcome).

## Results

The participants were mostly middle-aged (mean = 52.84 ± 8.15 years, range = 35 to 65 years). The proportions of women and men were similar (females = 51.9%, males = 48.1%). 16.9% of the participants were screened positive for depression, 15.1% were screened positive for anxiety. The majority of participants had rather high education (Level-1 = 19.8%, Level-2 = 21.5%, Level-3 = 17.9%, Level-4 = 40.6%).

Table 1 depicts median, mean, and standard deviation values of the valence and arousal ratings, indicating that the words and word categories were well separated in the valence and arousal spaces. Mean arousal ratings of neutral words were lower compared to unpleasant and pleasant words, for which mean arousal ratings were similar. The mean valence ratings showed a steady increase (from unpleasant through neutral to pleasant) and were more homogeneous across categories (see also Additional file 1: Figures S1 and S2). The valence ratings tended to be less variable compared to the arousal ratings. For comparison purposes, Additional file 1: Table S1 provides mean valence and arousal ratings for overlapping nouns from the studies from Kissler et al. [46] and Kanske et al. [54, 69], both of which had also used nine-point bipolar valence and unipolar arousal scales (although these studies used a graphical rendering (SAM scale)).

**Table 1:** Measures of central tendency and variability of the valence and arousal ratings

	Valence			Arousal		
	Median	Mean	SD	Median	Mean	SD
Unpleasant words						
Schande (shame)	3	3.03	1.85	5	4.98	2.70
Hass (hate)	2	2.58	2.05	6	5.62	2.83
Leiche (corpse)	2	2.80	2.03	6	5.38	2.83
Neid (envy)	2	2.82	1.81	5	4.96	2.66
Average for unpleasant words	2	2.81	1.66	5	5.23	2.35
Neutral words						
Folie (foil)	5	5.17	1.17	1	2.24	1.82
Text (text)	5	5.67	1.48	3	3.55	2.32
Objekt (object)	5	5.29	1.22	2	3.09	2.17
Agentur (agency)	5	4.93	1.22	2	2.99	2.16
Average for neutral words	5	5.26	0.82	2	2.96	1.63
Pleasant words						
Lachen (laughter)	9	8.01	1.90	7	5.90	2.50
Hobby (hobby)	8	7.33	1.96	6	5.28	2.63
Ferien (holidays)	9	7.88	1.95	7	6.03	2.66
Genuss (relish)	8	7.52	1.95	6	5.66	2.60
Average for pleasant words	9	7.68	1.72	6	5.72	2.27

Bipolar valence scale (1 = “very unpleasant”, 5 = “neither unpleasant nor pleasant”, 9 = “very pleasant”). Unipolar arousal scale (1 = “not arousing”, 9 = “very arousing”)

Table 2 summarizes the full linear model quality metrics for the six different outcomes. R^2^ values varied between 1.3% (valence by unpleasant words) and 5.4% (arousal by unpleasant words), and were higher in the arousal compared to the valence dimension. Notably, all three models in the valence dimension failed to reach statistical significance at *p* < 0.05, indicating that the valence ratings were unaffected by the selected predictors. Therefore, we refrain from interpreting any effects in the valence dimension.

**Table 2:** Model quality metrics for the six different outcomes

Outcome	R^2^	95% CI	*p* value
Valence			
Unpleasant words	0.013	0; 0.019	0.471
Neutral words	0.018	0; 0.028	0.207
Pleasant words	0.017	0; 0.026	0.243
Arousal			
Unpleasant words	0.054	0.015; 0.076	< 0.001
Neutral words	0.046	0.010; 0.066	< 0.001
Pleasant words	0.035	0.003; 0.052	0.004

The full model included the four explanatory variables of interest (i.e. age, sex, depression, and anxiety) and the two adjusted variables (i.e. education and examiner). N = 704, residual degrees of freedom = 693

In case of arousal by unpleasant words, there were significant effects of sex (df = 1, F = 5.325, *p* = 0.021), education (df = 3, F = 8.185, *p* < 0.001), and examiner (df = 3, F = 2.999, *p* = 0.030). The remaining effects were not significant (age: df = 1, F = 2.537, *p* = 0.111; depression: df = 1, F = 0.138, *p* = 0.709; anxiety: df = 1, F = 1.837, *p* = 0.175).

In case of arousal by neutral words, there were significant effects of age (df = 1, F = 17.747, *p* < 0.001) and anxiety (df = 1, F = 6.299, *p* = 0.012), and a trend effect of sex (df = 1, F = 3.407, *p* = 0. 065). The remaining effects were not significant (depression: df = 1, F = 0.428, *p* = 0.512; education: df = 3, F = 1.063, *p* = 0.363; examiner: df = 3, F = 1.393, *p* = 0.243).

In case of arousal by pleasant words, there was a trend effect of sex (df = 1, F = 3.758, *p* = 0.052) and a significant effect of examiner (df = 3, F = 4.587, *p* = 0.003). The remaining effects were not significant (age: df = 1, F = 0.044, *p* = 0.832; depression: df = 1, F = 0.347, *p* = 0.555; anxiety: df = 1, F = 2.196, *p* = 0.138; education: df = 3, F = 1.723, *p* = 0.160). Table 3 summarizes relevant linear model parameters for the explanatory variables of interest. The (generalized) variance inflation factors for all explanatory variables were close to 1 (age: 1.091, df = 1; sex: 1.069, df = 1; depression: 1.204, df = 1; anxiety: 1.222, df = 1; education: 1.144, df = 3; examiner: 1.026, df = 3), indicating that there was no problem with multi-collinearity.

**Table 3:** Model parameters for the four explanatory variables of interest stratified by outcome

Outcome	Explanatory variables of interest	Estimate^a^	95% CI	*p* value
Valence				
Unpleasant words (n.s.)	Age	− 0.001	− .017; 0.014	0.857
	Sex (female)	− .241	− .497; 0.013	0.063
	Depression	− .176	− .540; 0.187	0.340
	Anxiety	0.068	− .311; 0.449	0.721
Neutral words (n.s.)	Age	0.009	0.001; 0.017	0.020
	Sex (female)	− .009	− .134; 0.115	0.879
	Depression	− .068	− .246; 0.110	0.453
	Anxiety	0.123	− .062; 0.310	0.192
Pleasant words (n.s.)	Age	− .001	− .018; 0.014	0.812
	Sex (female)	0.261	− .002; 0.525	0.052
	Depression	0.021	− .354; 0.398	0.909
	Anxiety	− .362	− .755; 0.030	0.070
Arousal				
Unpleasant words	Age	0.017	− .004; 0.039	0.111
	Sex (female)	0.414	0.061; 0.767	0.021
	Depression	− .095	− .597; 0.407	0.709
	Anxiety	0.362	− .162; 0.887	0.175
Neutral words	Age	0.033	0.017; 0.048	< 0.001
	Sex (female)	− .232	− .478; 0.014	0.065
	Depression	0.117	− .234; 0.468	0.512
	Anxiety	0.469	0.102; 0.836	0.012
Pleasant words	Age	− .002	− .023; 0.019	0.832
	Sex (female)	− .338	− .681; − .004	0.052
	Depression	− .146	− .635; 0.341	0.555
	Anxiety	0.385	− .125; 0.895	0.138

All analyses were adjusted for education and examiner

^a^Non-standardized regression coefficient. The linear models in the valence dimension were not significant; effects of predictors in the valence dimension should not be interpreted

Table 4 comprises proportions of variance explained by groups of explanatory variables for the different outcomes. Notably, in the arousal dimension, in the neutral word category the explanatory variables as a group explained about 81% of the variance explained by the full model.

**Table 4:** Proportions of explained variance stratified by outcome

Outcome	Variance explained by full model^a^ (R^2^)	Variance explained by predictors of interest^b^
Valence		
Unpleasant words (n.s.)	0.0138	0.0068
Neutral words (n.s.)	0.0189	0.0093
Pleasant words (n.s.)	0.0180	0.0095
Arousal		
Unpleasant words	0.0544	0.0140
Neutral words	0.0462	0.0375
Pleasant words	0.0359	0.0080

^a^Included age, sex, depression, anxiety, education, and examiner

^b^Age, sex, depression, and anxiety. The linear models in the valence dimension were not significant

**Fig. 2:**
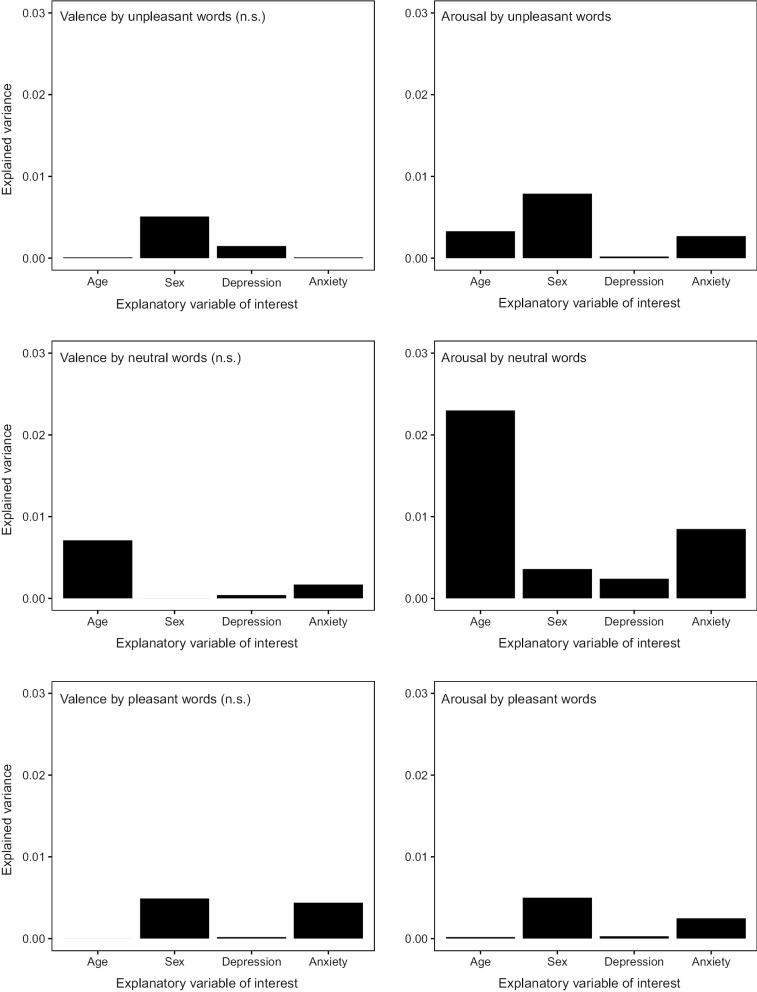
Absolute proportions of variance explained by the four explanatory variables of interest, stratified by outcome. The linear models in the valence dimension were not significant

Figure 2 displays proportions of variance explained by individual explanatory variables of interest, i.e. their relative importance.

We computed several sensitivity analyses in order to further investigate the robustness of the (trend) effects that were found in the arousal dimension in the main analyses. First of all, given that the valence ratings of the words had been given strictly prior to the arousal ratings of the words, there is a possibility that the valence ratings may have affected the effects of the explanatory variables of interest on the arousal ratings. To assess this possibility, we re-computed the linear regression analyses in the arousal dimension, thereby additionally adjusting for the average valence rating in a given word category. All three arousal models were significant (unpleasant: R^2^ = 0.121; *p* = < 0.001; neutral: R^2^ = 0.066; *p* = < 0.001; pleasant: R^2^ = 0.133; *p* = < 0.001). The (trend) effects of sex that were found in the main analyses for all three word categories as well as the effects of age and anxiety found for arousal by neutral words (Table 2) were also found under additional adjustment for valence rating (Additional file 1: Table S2). Most importantly, the sizes and directions of effects were largely comparable to the effects found in the main analyses.

Second, given that the words in the unpleasant and pleasant categories consisted of a mixture of *emotion-terms* (unpleasant: Schande (shame), Hass (hate), Neid (envy); pleasant: Lachen (laughter), Genuss (relish)) on the one hand and *emotion-laden terms* (unpleasant: Leiche (corpse); pleasant: Hobby (hobby), Ferien (holidays)) on the other hand, we re-computed the regression analyses for unpleasant and pleasant words separately for emotion-terms and emotion-laden terms. In the valence dimension, all four models failed to reach statistical significance (unpleasant emotion-terms: R^2^ = 0.014, *p* = 0.401; unpleasant emotion-laden terms: R^2^ = 0.012, *p* = 0.527; pleasant emotion-terms: R^2^ = 0.017, *p* = 0.261; pleasant emotion-laden terms: R^2^ = 0.018, *p* = 0.217), while all four arousal models were significant (unpleasant emotion-terms: R^2^ = 0.060, *p* < 0.001; unpleasant emotion-laden terms: R^2^ = 0.031, *p* = 0.015; pleasant emotion-terms: R^2^ = 0.039, *p* = 0.001; pleasant emotion-laden terms: R^2^ = 0.028, *p* = 0.025). The (trend) effects of sex that were found in the main arousal analyses for the unpleasant und pleasant categories (Table 2) were also found in both emotion-terms and emotion-laden terms separately (Additional file 1: Table S3). Most importantly, the sizes and directions of effects were largely comparable in the three analyses (i.e. all four words vs. emotion-terms only vs. emotion-laden terms only).

Third, to address the concern that participants may have applied certain response tendencies during rating the words, we further excluded (1) subjects who exhibited standard deviations of zero across either valence or arousal ratings, demonstrating that these subjects had rated all 12 words identically. We additionally excluded (2) subjects who had rated all four words of a given word category with one of the following valence-arousal rating combinations: 5–5, 1–1, or 9–1. For instance, a rating combination of 1–1 (or 9–1) would indicate that a word was perceived as extremely unpleasant (or pleasant) and completely non-arousing at the same time. A rating combination of 5–5 would indicate that a word was perceived as completely neutral and comparably highly arousing (i.e. almost as arousing as the unpleasant or pleasant words were perceived on average; cf. Table 1) at the same time. Due to the presence of the three distinct word categories, such rating behavior may be indicative of either deficient task comprehension or non-compliance. These adjustments led to the exclusion of 99 additional participants. In the valence dimension, all three models failed to reach statistical significance (unpleasant: R^2^ = 0.025, *p* = 0.110; neutral: R^2^ = 0.017, *p* = 0.368; pleasant: R^2^ = 0.022, *p* = 0.204), while all arousal models were significant (unpleasant: R^2^ = 0.061, *p* < 0.001; neutral: R^2^ = 0.060, *p* < 0.001; pleasant: R^2^ = 0.033, *p* = 0.026). The (trend) effects of sex that were found in all word categories in the arousal dimension in the main analyses (Table 2) were found here only in case of the unpleasant words. The effects of age and anxiety that were found in case of arousal by neutral words in the main analyses were also found here (Additional file 1: Table S4). The magnitudes and directions of the effects reproduced here largely correspond to the effects found in the main analyses.

## Discussion

In order to assess the feasibility of obtaining and determining the structure of valence and arousal ratings in a large, population-based, observational, epidemiologic study, the analyses reported here explored the relative importance of four variables of interest (age, sex, depression, and anxiety) for valence and arousal ratings of a small number of pre-selected unpleasant, neutral, and pleasant words. The variables were selected based on previous studies which indicated associations of those variables with affective quality perception or experience, established examples of which include an age-related positivity effect [13], increased emotion recognition capabilities of women [19], and dysfunctional affective experience and perception during states of depression or anxiety [42]. Valence and arousal ratings were employed, because these two dimensions represent essential qualities of affective experience and affective quality perception [4, 10]. Written words were used as stimuli, because they provide easy and unobtrusive access to the human affect interface [70]. Moreover, words enable the control for certain psycholinguistic measures known to influence cognitive processing [26].

Compared to previous valence and arousal investigations, the present study is characterized by certain features, which have the potential to extend existing findings. First and foremost, while previous studies had most often taken the form of laboratory experiments, the present study utilized the setting of a large, population-based, observational, epidemiologic study, the BiDirect Study [61]. Second, while previous studies often included rather small samples, the present study analyzed ratings of more than 700 participants. Third, while previous studies usually included young (often predominantly female) university students, the BiDirect Study had recruited a sex-balanced sample of middle-aged to elderly adults randomly invited from the general population. Finally, while previous studies had usually focused on only one or two variables of interest, the present study explored the roles of four relevant explanatory variables.

From our point of view, the rating patterns observed in the present study (Table 1, Additional file 1: Figures S1 and S2) indicated that it was feasible to obtain valence and arousal rating data of acceptable quality in the context of the BiDirect Study. Taking into account existing methodological differences (e.g. the number of words to be rated, the rating context, or the underlying population), this view is further supported by the comparison of the present rating results with ratings from previous studies [46, 54] for overlapping nouns (Additional file 1: Table S1). The ratings obtained in the present study consistently fall in between those from Kissler et al. [46] and Kanske et al. [54] and exhibit similar variances, despite large heterogeneity of the rating population. However, the predictors used in the present study systematically explained only very little variance in the data, with significant effects occurring only for arousal, but not for valence.

There is an ongoing debate regarding the relationship between valence and arousal, and it is currently unresolved whether valence is better represented by a bipolar or by two unipolar dimensions [4, 7, 8, 12]. However, it can be stated that the average valence and arousal ratings collected here (Table 1; Additional file 1: Figures S1, S2) exhibited the expected distributions with regard to the pre-defined word categories. Moreover, the mean ratings appear to be rather stable (Table 1). Thus, altogether the present results do not argue against a further pursuit of emotional (affective) epidemiology [71]. This may be worthwhile given that emotions and affect matter for health and health perception [72]: frequent or chronic experience of negative emotions (e.g. sadness, fear) and affect may influence the development of somatic conditions such as e.g. infectious disease [73] or lung dysfunction [74] and may exacerbate chronic somatic diseases such as diabetes [75], arthritis [76], cancer [77], and cardiovascular disease [78]. Frequent experience of positive emotions (e.g. enjoyment) and affect, in contrast, likely exert protective effects on health [79], e.g. by reducing the risk of developing cardiovascular disease [80].

The present study found that the proportions of variance explained by the specific linear model applied (which included the predictors age, sex, depression, and anxiety, plus the adjusted variables education and examiner) were in a range between 1.3 and 5.4% and thus very low. In the valence dimension, the model even failed to reach significance for each of the three words categories (due to this finding, we refrain from interpreting any effects regarding the valence dimension). This indicates that the valence ratings were largely unaffected by the selected predictors, and that there may be other, potentially more relevant variables that were not included in the present study. Besides the inherent affective properties of the words themselves, likely candidates include situational, personality-, language-, or culture-related factors.

The modeling results regarding the arousal ratings for the three word categories showed small effects, which were slightly larger for unpleasant (5.4% explained variance) and neutral (4.6%) compared to pleasant (3.5%) words. Interestingly, the explanatory variables of interest as a group tended to explain the most variance in case of arousal by *neutral* words (Table 4, Fig. 2). This could be taken as a hint that the arousal dimension of affective quality perception, especially if accessed by rather neutral (or possibly “ambiguous”) stimuli, may be comparably well-suited to detect subtle inter-individual affective tendencies or differences.

Figure 2 (right column) displays that the relative importance *patterns* (i.e. the degrees of variance explained by the four explanatory variables of interest relative to each other) qualitatively differed between the three word categories in the arousal dimension. Thus, the very same variables tended to explain more or less variance depending on how a group of people had pre-categorized the utilized word stimuli (unpleasant, neutral, or pleasant). This impression is corroborated by the statistical effects regarding the individual predictors.

In the present study, (trend) effects of sex on arousal ratings were found for all word categories (Table 2). In case of unpleasant words, female sex was associated with *in*creased arousal. This effect was also detected in all sensitivity analyses (Additional file 1: Tables S2, S3, and S4). In case of neutral and pleasant words, female sex was associated with *de*creased arousal. Noteworthy, the latter effects were not found in the third sensitivity analysis (Additional file 1: Table S4), where additional (possibly non-compliant) participants had been excluded.

Sex has previously been found to influence affective perception [27] and experience [20, 81–84]. Previous findings indicated that women are possibly more emotionally reactive and receptive. Moreover, sex-related differences were more consistently observed with respect to unpleasant emotions [85, 86]. This is in line with the present result that the sex effect on arousal ratings seemed to be most consistent in case of the unpleasant words. Furthermore, sex-specificity during emotional processing was reflected in a tendency of women to “prefer” unpleasant stimuli, which was in contrast to a preference for pleasant stimuli in men [87]. This is in line with the present finding that women rated the unpleasant words to be more arousing, but the pleasant (and neutral) words to be less arousing compared to men.

More specifically, previous findings regarding effects of sex on arousal ratings of words were mixed [26]. Redondo et al. [51] and Gilet et al. [24] did not find any sex-related differences. Soares et al. [28] found that women gave higher mean arousal ratings overall. Partly in line with the present results, Grunwald et al. [27] found that women gave higher mean arousal ratings for pleasant and particularly for unpleasant words. Also partly in line with the present results, Söderholm et al. [26] found that women rated unpleasant and neutral words as more arousing compared to men.

In the present study, an effect of age on arousal ratings was found for the neutral words (Table 2). Older age was associated with increased arousal ratings. This effect was also detected in all applicable sensitivity analyses (Additional file 1: Tables S2, S4). Moreover, this effect was the statistically most robust effect among effects of individual predictors of interest.

Aging has previously been found to influence affective perception [27] and experience, whereat the age-related positivity effect [81, 82, 88] is one prominent example. Findings regarding an influence of age on arousal ratings of words were again mixed, which may at least partly be due to methodological differences between studies [26]. Using a SAM scale, Keil and Freund [25] found that older adults gave higher arousal ratings for unpleasant words compared to young adults. However, using a seven-point Likert scale (“very relaxed” to “very tensed”), Grühn and Smith [23] found that older adults gave lower arousal ratings for unpleasant words and higher arousal ratings for pleasant words compared to young adults. Using a seven-point Likert scale (“very calming” to “very arousing”), Söderholm et al. [26] reported complex results. Using a seven-point Likert scale (“very relaxed” to “very tensed”), Gilet et al. [24] found that middle-aged and older adults gave higher mean arousal ratings compared to young adults; this finding is basically in line with the results of the present study. Eventually, using a six-point Likert scale (“not at all intense” to “very intense”), Grunwald et al. [27] found no age-related differences in arousal ratings for unpleasant or pleasant words. However, in line with the findings of the present study, older adults gave higher arousal ratings compared to young or middle-aged adults for neutral words.

In the present study, age was modeled as a continuous variable, enabling extrapolation: for instance, the effect size point estimate for age observed here (0.033; Table 2) indicates that on average, holding the other variables in the model constant, becoming 30 years older (at least in the age range covered) would go along with an increase in average arousal ratings of neutral words of one point on the nine-point scale applied here.

In the present study, an effect of anxiety on arousal ratings was found for the neutral words (Table 2): anxiety was associated with increased arousal. This effect was also found in all applicable sensitivity analyses (Additional file 1: Tables S2, S4). Notably, no effects of depression on arousal ratings were found in any of the word categories in any of the analyses (Table 2; Additional file 1: Tables S2, S3, and S4).

The literature regarding influences of anxiety on arousal ratings of words is sparse. A study by Kanske et al. [89] probed the influence of depression and anxiety on valence and arousal ratings of auditorily presented words. The results indicated that higher anxiety scores were associated with higher arousal ratings of unpleasant words (r = 0.43). In line with the results of the present study, which showed a positive association between anxiety and arousal by neutral words (Table 2), the correlation between anxiety scores and arousal ratings of neutral words reported in Kanske et al. [89] was also positive (r = 0.11; however, this correlation was not significant).

Taken together, the results of the present study indicated that across the three word categories, with respect to the predictors of interest that were explored here, it seems that sex tended to be important in principle, while the importance of age and anxiety was specific for non-toned (i.e. neutral) information. Noteworthy, anxiety tended to explain more variance than depression (Fig. 2), although both were binary variables. However, this finding should be interpreted with caution, bearing in mind the strong comorbidity of depression and anxiety [90], a reality which was also reflected by the association between the depression and anxiety predictor variables (Additional file 1: Figure S3). However, the number of participants who fell into respective categories (depression only: N = 62; anxiety only: N = 52; both depression and anxiety: N = 55) was very similar, and the age and sex distributions were largely comparable between categories. Thus, the finding that anxiety stood up to depression in the present context of an affective quality perception task may be informative.

### Strengths, limitations, and future directions

In the present study, a large, sex-balanced sample of middle-aged to elderly participants, who were randomly invited from the general population of a mid-sized, north-western European university city, was analyzed. Overcoming small and often sex-dysbalanced participant samples of previous studies, the present study investigated valence and arousal ratings of unpleasant, neutral, and pleasant German nouns and simultaneously explored the relative importance of four relevant variables with regard to six relevant outcomes of affective quality perception along those dimensions. However, certain limitations need to be considered. First, given the observational data, unmeasured or residual confounding cannot be ruled out. Second, the very low proportions of explained variance suggest that there may be other, potentially more relevant explanatory variables in addition to those considered here. Candidates include e.g. certain personality dimensions, most likely extraversion and neuroticism [4, 11]. Third, potential data acquisition problems (deficient task comprehension, non-compliance) might have, in addition to idiographic variability, contributed to observed “deviant” ratings and non-monotonic frequency distributions. Moreover, the data were collected exclusively in a Western cultural context. Furthermore, the perceived communicative context, which was not considered here, may play an important role (e.g. [91, 92]). A further limitation to age as a predictor is that the sample did not include young adults. Finally, the words used here as stimuli are somewhat heterogeneous, as they include both emotion-terms (i.e. hate, envy) as well as emotion-laden terms (e.g. corpse, holidays). Previous research has shown processing differences between those two categories (e.g. [93]) that might extend to processing malleability by individual differences [94]. With regard to future directions, it is noteworthy that follow-up studies could use more, more frequent, less heterogeneous, and less abstract word stimuli and an implicit instead of an explicit task (e.g. the so-called emotional stroop paradigm [95]).

## Conclusion

The present results show that it is possible to perform valence and arousal ratings outside the laboratory in the setting of large epidemiologic studies, bearing acceptable data quality. Overall, mean ratings replicated those previously obtained in laboratory settings. While effects of the selected predictor variables were generally weak, the results indicate that the relative importance patterns regarding age, sex, depression, and anxiety differed for arousal ratings between three different word categories. Overall, particularly sex, but also age and anxiety (particularly with respect to neutral (or “ambiguous”) material) tended to be more important, while depression appeared less important.

## Supplementary information

**Additional file 1: Table S1**. Valence and arousal ratings for overlapping words as mentioned in Kissler et al. (1) and as published by Kanske et al. (2,3). **Table S2**. Sensitivity analysis - additional adjustment for valence rating. **Table S3**. Sensitivity analysis - emotion-terms only vs. emotion-laden terms only. **Table S4**. Sensitivity analysis - participants exhibiting hints for deficient task comprehension or non-compliance (N = 99) were excluded. **Figure S1**. Distributions (boxplots) of the average valence (A) and arousal ratings (B) stratified by word category (unpleasant vs. neutral vs. pleasant). Grayish dots denote individual data points (jittered). Thick black dots denote means. **Figure S2**. Left panel: Frequency distributions (heatmaps) of combined raw valence and arousal ratings stratified by word category (in rows; unpleasant vs. neutral vs. pleasant). Right panel: Distributions (scatterplots) of individual combined average valence and arousal ratings stratified by word category. **Figure S3**. Mosaic plot depicting the association between the two explanatory variables of interest “depression” and “anxiety”. N total = 704. Depression = “Yes” and Anxiety = “No”: N = 62. Anxiety = “Yes” and Depression = “No”: N = 52. Depression = “Yes” and Anxiety = “Yes”: N = 55. Depression = “No” and Anxiety = “No”: N = 535.

## Data Availability

The data of the BiDirect Study, including the data used and/or analysed during the current study, are available via the last author on reasonable request.
