# Supplementary material for: Investigating the roles of age, sex, depression, and anxiety for valence and arousal ratings of words: a population-based study
Source: BMC Psychol. 2020 Nov 7;8:118. doi: 10.1186/s40359-020-00485-3 (PMC7648958; doi:10.1186/s40359-020-00485-3)
Supplement: Supplementary file 1 — Additional file 1: Table S1. Valence and arousal ratings for overlapping words as mentioned in Kissler et al. (1) and as published by Kanske et al. (2,3). Table S2. Sensitivity analysis - additional adjustment for valence rating. Table S3. Sensitivity analysis - emotion-terms only vs. emotion-laden terms only. Table S4. Sensitivity analysis - participants exhibiting hints for deficient task comprehension or non-compliance (N = 99) were excluded. Figure S1. Distributions (boxplots) of the average valence (A) and arousal ratings (B) stratified by word category (unpleasant vs. neutral vs. pleasant). Grayish dots denote individual data points (jittered). Thick black dots denote means. Figure S2. Left panel: Frequency distributions (heatmaps) of combined raw valence and arousal ratings stratified by word category (in rows; unpleasant vs. neutral vs. pleasant). Right panel: Distributions (scatterplots) of individual combined average valence and arousal ratings stratified by word category. Figure S3. Mosaic plot depicting the association between the two explanatory variables of interest “depression” and “anxiety”. N total = 704. Depression = “Yes” and Anxiety = “No”: N = 62. Anxiety = “Yes” and Depression = “No”: N = 52. Depression = “Yes” and Anxiety = “Yes”: N = 55. Depression = “No” and Anxiety = “No”: N = 535. [file 40359_2020_485_MOESM1_ESM.docx]

**Additional file 1: Table S1.** Valence and arousal ratings for overlapping words as mentioned in Kissler et al. (1) and as published by Kanske et al. (2,3).

|  | **Kissler et al.** | | | | **Kanske et al.** | | | |
| --- | --- | --- | --- | --- | --- | --- | --- | --- |
|  | **Valence** | | **Arousal** | | **Valence** | | **Arousal** | |
| **Unpleasant words** | **Mean** | **SD** | **Mean** | **SD** | **Mean** | **SD** | **Mean** | **SD** |
| Schande (shame) | 2.45 | 1.21 | 5.45 | 2.40 | 3.52 | 2.37 | 5.39 | 2.32 |
| Hass (hate) | 2.05 | 0.97 | 5.82 | 1.94 | 3.14 | 2.54 | 7.40 | 2.03 |
| Leiche (corpse) | 2.58 | 0.94 | 5.47 | 1.76 | 3.03 | 2.79 | 7.18 | 2.09 |
| Neid (envy) | 2.41 | 1.74 | 5.45 | 2.32 | 3.49 | 2.48 | 5.85 | 2.10 |
| **Neutral words** |  |  |  |  |  |  |  |  |
| Folie (foil) | 5.08 | 0.68 | 2.08 | 1.47 | 5.07 | 0.74 | 2.15 | 1.59 |
| Text (text) | 5.50 | 1.10 | 2.58 | 1.79 | ---^#^ | --- | --- | --- |
| Objekt (object) | 5.00 | 0 | 1.90 | 1.66 | --- | --- | --- | --- |
| Agentur (agency) | 4.75 | 0.94 | 3.08 | 1.95 | --- | --- | --- | --- |
| **Pleasant words** |  |  |  |  |  |  |  |  |
| Lachen (laughter) | 8.20 | 0.83 | 6.08 | 2.18 | 7.12 | 2.78 | 6.23 | 2.53 |
| Hobby (hobby) | 7.62 | 1.17 | 4.95 | 2.27 | 6.25 | 1.98 | 4.12 | 2.75 |
| Ferien (holidays) | 8.17 | 0.81 | 5.11 | 2.09 | --- | --- | --- | --- |
| Genuss (relish) | 8.08 | 0.71 | 4.33 | 2.37 | 6.46 | 2.63 | 6.45 | 2.26 |

Note. Both Kissler et al. and Kanske et al. used a bipolar nine-point SAM valence scale and a unipolar nine-point SAM arousal scale. ^#^Word not included in database. N = 704.

**Additional file 1: Table S2.** Sensitivity analysis - additional adjustment for valence rating.

| **Arousal by** | **Predictor of interest** | **Estimate*^a^*** | **95% CI** | **p-value** |
| --- | --- | --- | --- | --- |
| Unpleasant words | Age | 0.017 | -0.003; 0.038 | 0.109 |
|  | Sex (female) | 0.325 | -0.015; 0.666 | 0.061 |
|  | Depression | -0.160 | -0.645; 0.324 | 0.516 |
|  | Anxiety | 0.387 | -0.118; 0.894 | 0.133 |
| Neutral words | Age | 0.030 | 0.015; 0.045 | < 0.001 |
|  | Sex (female) | -0.229 | -0.473; 0.015 | 0.065 |
|  | Depression | 0.136 | -0.211; 0.484 | 0.441 |
|  | Anxiety | 0.434 | 0.070; 0.798 | 0.019 |
| Pleasant words | Age | -0.001 | -0.021; 0.018 | 0.886 |
|  | Sex (female) | -0.446 | -0.772; -0.120 | 0.007 |
|  | Depression | -0.155 | -0.619; 0.307 | 0.509 |
|  | Anxiety | 0.534 | 0.049; 1.020 | 0.030 |

Note. All analyses were adjusted for education, examiner, and valence rating. *^a^*Non-standardized regression coefficient. N = 704.

**Additional file 1: Table S3.** Sensitivity analysis - emotion-terms only vs. emotion-laden terms only.

|  |  | **Emotion-terms only** | | | | **Emotion-laden terms only** | | | |
| --- | --- | --- | --- | --- | --- | --- | --- | --- | --- |
| **Outcome** | **Predictor of interest** | **Estimate*^a^*** | **95% CI** | **p-value** | **Estimate*^a^*** | | **95% CI** | **p-value** |  |
| **Valence** |  |  |  |  |  | |  |  |  |
| Unpleasant words (n.s.) | Age | -0.000 | -0.016; 0.015 | 0.923 | -0.003 | | -0.022; 0.016 | 0.729 |  |
|  | Sex (female) | -0.254 | -0.513; 0.003 | 0.053 | -0.201 | | -0.515; 0.111 | 0.206 |  |
|  | Depression | -0.220 | -0.588; 0.148 | 0.241 | -0.046 | | -0.492; 0.400 | 0.838 |  |
|  | Anxiety | 0.067 | -0.317; 0.452 | 0.729 | 0.072 | | -0.394; 0.538 | 0.761 |  |
| Pleasant words (n.s.) | Age | -0.004 | -0.021; 0.012 | 0.576 | 0.000 | | -0.016; 0.018 | 0.921 |  |
|  | Sex (female) | 0.282 | 0.009; 0.556 | 0.042 | 0.240 | | -0.035; 0.515 | 0.087 |  |
|  | Depression | 0.094 | -0.294; 0.483 | 0.633 | -0.050 | | -0.443; 0.341 | 0.799 |  |
|  | Anxiety | -0.403 | -0.809; 0.003 | 0.052 | -0.321 | | -0.731; 0.088 | 0.123 |  |
| **Arousal** |  |  |  |  |  | |  |  |  |
| Unpleasant words | Age | 0.020 | -0.001; 0.043 | 0.069 | 0.009 | | -0.017; 0.035 | 0.499 |  |
|  | Sex (female) | 0.372 | 0.012; 0.731 | 0.042 | 0.541 | | 0.112; 0.971 | 0.013 |  |
|  | Depression | -0.154 | -0.666; 0.357 | 0.553 | 0.082 | | -0.529; 0.693 | 0.792 |  |
|  | Anxiety | 0.467 | -0.067; 1.003 | 0.086 | 0.046 | | -0.593; 0.685 | 0.887 |  |
| Pleasant words | Age | -0.002 | -0.024; 0.018 | 0.806 | -0.001 | | -0.025; 0.021 | 0.872 |  |
|  | Sex (female) | -0.358 | -0.706; -0.010 | 0.043 | -0.318 | | -0.689; 0.052 | 0.092 |  |
|  | Depression | -0.079 | -0.575; 0.416 | 0.752 | -0.213 | | -0.742; 0.314 | 0.427 |  |
|  | Anxiety | 0.301 | -0.216; 0.819 | 0.253 | 0.468 | | -0.083; 1.021 | 0.096 |  |

Note. All analyses were adjusted for education and examiner. *^a^*Non-standardized regression coefficient. The linear models in the valence dimension were not significant; effects of predictors in the valence dimension should not be interpreted. N = 704.

**Additional file 1: Table S4.** Sensitivity analysis - participants exhibiting hints for deficient task comprehension or non-compliance (N = 99) were excluded.

| **Outcome** | **Predictor of interest** | **Estimate*^a^*** | **95% CI** | **p-value** |
| --- | --- | --- | --- | --- |
| **Valence** |  |  |  |  |
| Unpleasant words (n.s.) | Age | -0.001 | -0.017; 0.014 | 0.817 |
|  | Sex (female) | -0.370 | -0.627; -0.112 | 0.004 |
|  | Depression | -0.147 | -0.509; 0.215 | 0.425 |
|  | Anxiety | -0.029 | -0.409; 0.351 | 0.880 |
| Neutral words (n.s.) | Age | 0.006 | -0.002; 0.014 | 0.152 |
|  | Sex (female) | -0.032 | -0.165; 0.100 | 0.630 |
|  | Depression | -0.069 | -0.257; 0.117 | 0.466 |
|  | Anxiety | 0.181 | -0.015; 0.378 | 0.070 |
| Pleasant words (n.s.) | Age | -0.008 | -0.024; 0.008 | 0.344 |
|  | Sex (female) | 0.319 | 0.049; 0.590 | 0.020 |
|  | Depression | -0.072 | -0.452; 0.307 | 0.707 |
|  | Anxiety | -0.209 | -0.608; 0.189 | 0.303 |
| **Arousal** |  |  |  |  |
| Unpleasant words | Age | 0.018 | -0.003; 0.040 | 0.100 |
|  | Sex (female) | 0.646 | 0.290; 1.002 | < 0.001 |
|  | Depression | -0.065 | -0.566; 0.434 | 0.796 |
|  | Anxiety | 0.361 | -0.164; 0.886 | 0.177 |
| Neutral words | Age | 0.035 | 0.019; 0.051 | < 0.001 |
|  | Sex (female) | -0.172 | -0.428; 0.082 | 0.184 |
|  | Depression | 0.119 | -0.240; 0.479 | 0.515 |
|  | Anxiety | 0.552 | 0.174; 0.930 | 0.004 |
| Pleasant words | Age | -0.004 | -0.025; 0.015 | 0.645 |
|  | Sex (female) | -0.171 | -0.503; 0.160 | 0.311 |
|  | Depression | -0.317 | -0.784; 0.149 | 0.182 |
|  | Anxiety | 0.496 | 0.005; 0.986 | 0.047 |

Note. All analyses were adjusted for education and examiner. *^a^*Non-standardized regression coefficient. The linear models in the valence dimension were not significant; effects of predictors in the valence dimension should not be interpreted. N = 605.

**Additional file 1: Figure S1.** Distributions (boxplots) of the average valence (A) and arousal ratings (B) stratified by word category (unpleasant vs. neutral vs. pleasant). Grayish dots denote individual data points (jittered). Thick black dots denote means.

**
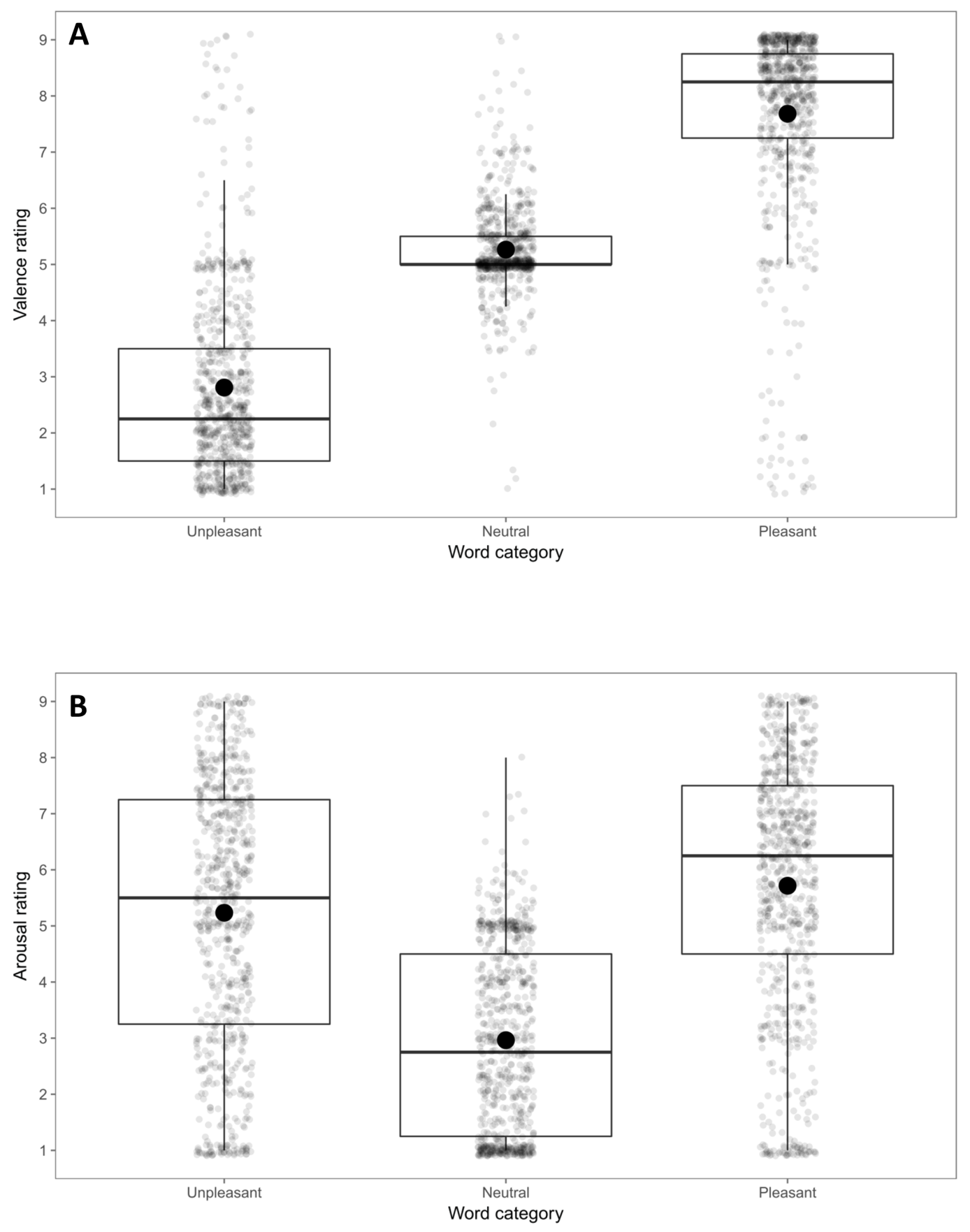
**

**Additional file 1: Figure S2.** Left panel: Frequency distributions (heatmaps) of combined *raw* valence and arousal ratings stratified by word category (in rows; unpleasant vs. neutral vs. pleasant). Right panel: Distributions (scatterplots) of individual combined *average* valence and arousal ratings stratified by word category.


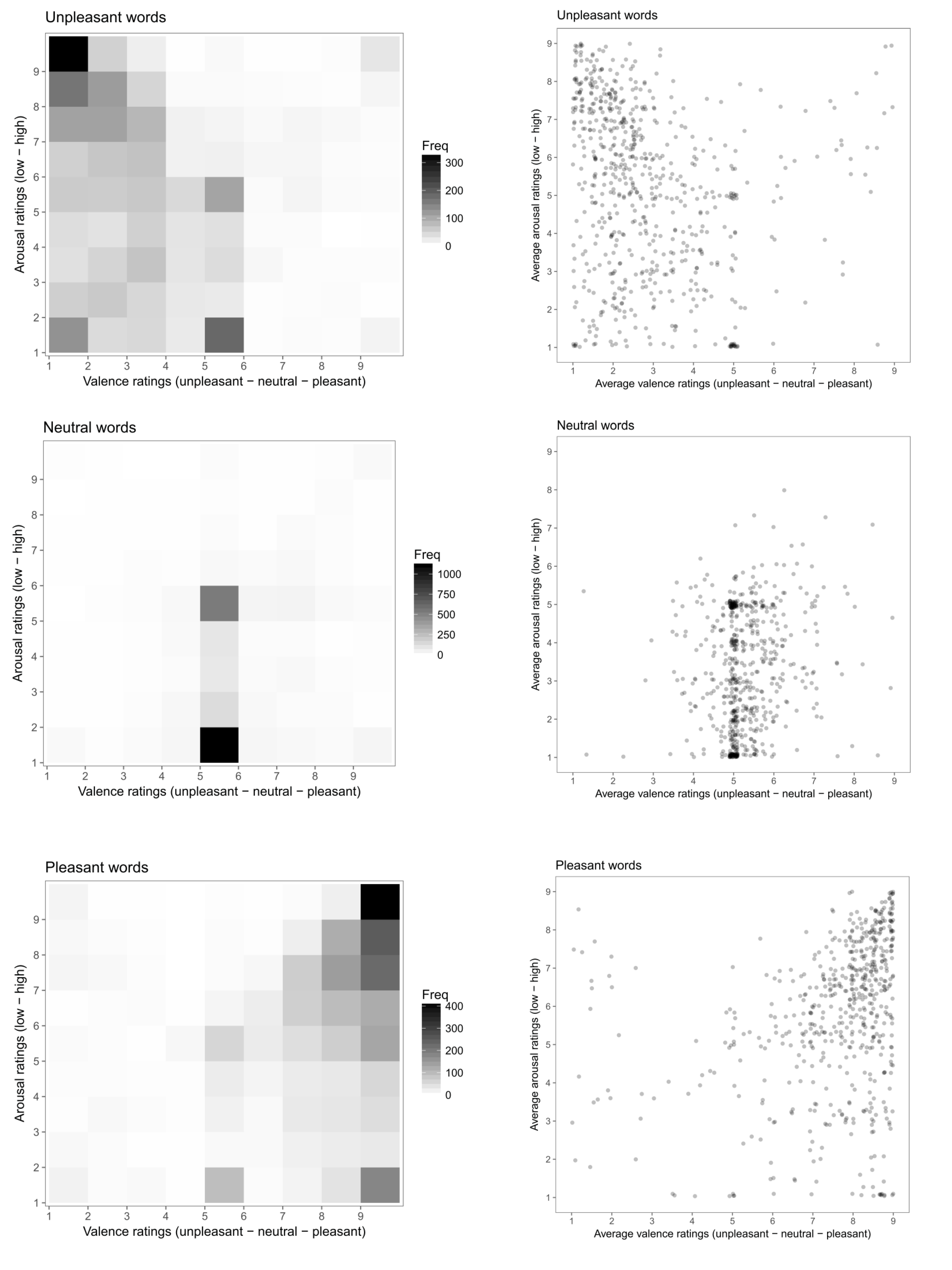


**Additional file 1: Figure S3.** Mosaic plot depicting the association between the two explanatory variables of interest “depression” and “anxiety”. N total = 704. Depression = “Yes” and Anxiety = “No”: N = 62. Anxiety = “Yes” and Depression = “No”: N = 52. Depression = “Yes” and Anxiety = “Yes”: N = 55. Depression = “No” and Anxiety = “No”: N = 535.


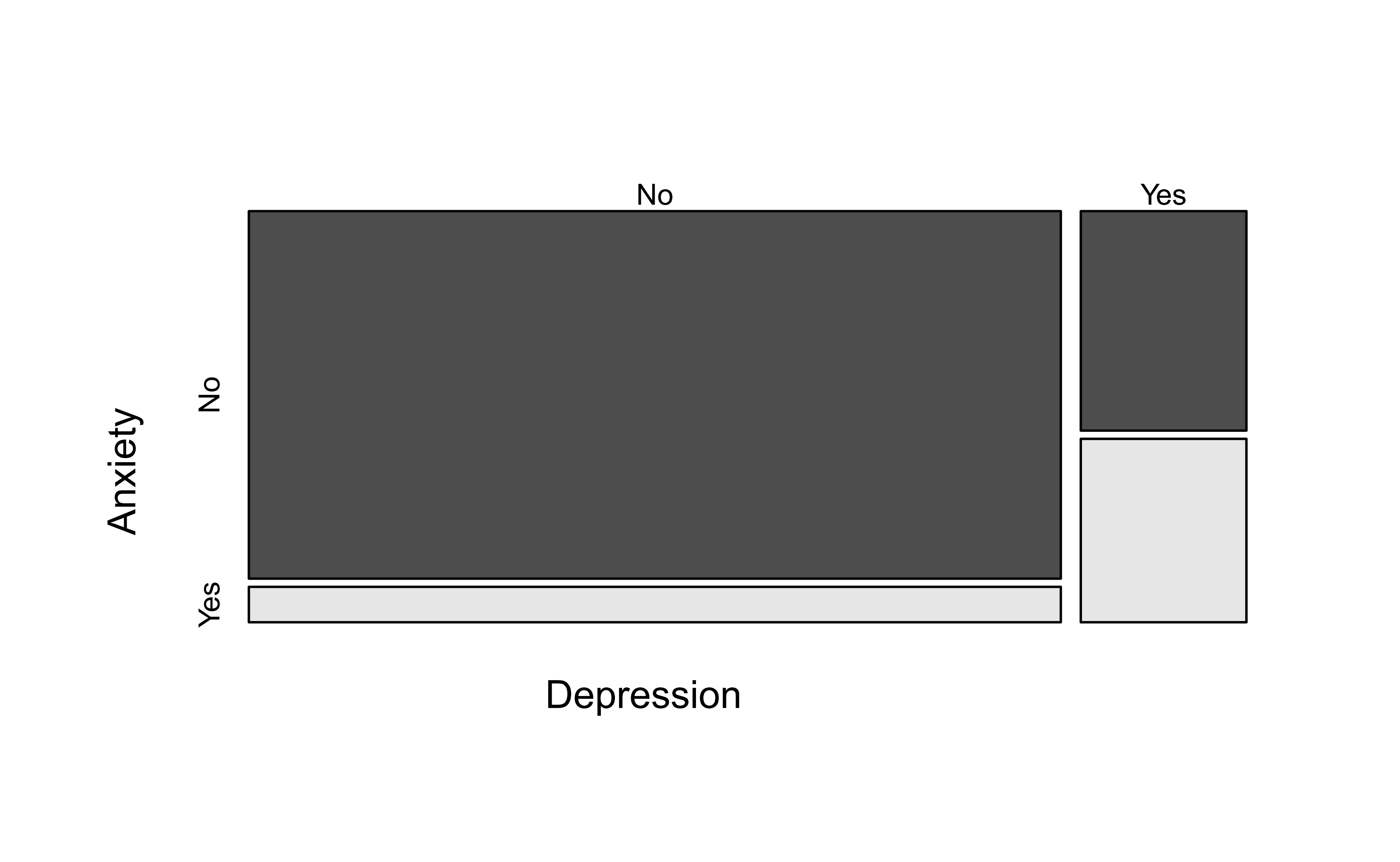


**References**

1. Kissler J, Herbert C, Peyk P, Junghofer M. Buzzwords: early cortical responses to emotional words during reading. Psychol Sci [Internet]. 2007 Jun [cited 2015 Aug 11];18(6):475–80. Available from: http://www.ncbi.nlm.nih.gov/pubmed/17576257

2. Kanske P, Kotz SA. Leipzig Affective Norms for German: A reliability study. Behav Res Methods. 2010;42(4):987–91.

3. Kanske P, Kotz SA. LANG_database.txt [Internet]. 2010 [cited 2020 Sep 4]. Available from: https://link.springer.com/article/10.3758/BRM.42.4.987
